# Blood-Based Biomarkers for Alzheimer’s Disease Diagnosis and Progression: An Overview

**DOI:** 10.3390/cells11081367

**Published:** 2022-04-17

**Authors:** Angelica Varesi, Adelaide Carrara, Vitor Gomes Pires, Valentina Floris, Elisa Pierella, Gabriele Savioli, Sakshi Prasad, Ciro Esposito, Giovanni Ricevuti, Salvatore Chirumbolo, Alessia Pascale

**Affiliations:** 1 Department of Biology and Biotechnology, University of Pavia, 27100 Pavia, Italy; 2 Almo Collegio Borromeo, 27100 Pavia, Italy; 3 Department of Internal Medicine and Therapeutics, University of Pavia, 27100 Pavia, Italy; adelaide.carrara01@universitadipavia.it (A.C.); valentina.floris01@universitadipavia.it (V.F.); 4 Department of Biological Sciences, Columbia University, New York, NY 10027, USA; vgp2107@columbia.edu; 5 School of Medicine, Faculty of Clinical and Biomedical Sciences, University of Central Lancashire, Preston PR1 2HE, UK; epierella1@uclan.ac.uk; 6 Emergency Department, IRCCS Policlinico San Matteo, 27100 Pavia, Italy; gabrielesavioli@gmail.com; 7 Faculty of Medicine, National Pirogov Memorial Medical University, 21018 Vinnytsya, Ukraine; sakshiprasad8@gmail.com; 8 Unit of Nephrology and Dialysis, ICS Maugeri, University of Pavia, 27100 Pavia, Italy; ciro.esposito@unipv.it; 9 Department of Drug Sciences, University of Pavia, 27100 Pavia, Italy; 10 Department of Neurosciences, Biomedicine and Movement Sciences, University of Verona, 37129 Verona, Italy; salvatore.chirumbolo@univr.it; 11 Department of Drug Sciences, Section of Pharmacology, University of Pavia, 27100 Pavia, Italy; alessia.pascale@unipv.it

**Keywords:** Alzheimer’s disease, biomarker, diagnosis, oxidative stress, gut microbiota, miRNA, lipid, vitamin, tau, amyloid-beta

## Abstract

Alzheimer’s Disease (AD) is a progressive neurodegenerative disease characterized by amyloid-β (Aβ) plaque deposition and neurofibrillary tangle accumulation in the brain. Although several studies have been conducted to unravel the complex and interconnected pathophysiology of AD, clinical trial failure rates have been high, and no disease-modifying therapies are presently available. Fluid biomarker discovery for AD is a rapidly expanding field of research aimed at anticipating disease diagnosis and following disease progression over time. Currently, Aβ_1–42_, phosphorylated tau, and total tau levels in the cerebrospinal fluid are the best-studied fluid biomarkers for AD, but the need for novel, cheap, less-invasive, easily detectable, and more-accessible markers has recently led to the search for new blood-based molecules. However, despite considerable research activity, a comprehensive and up-to-date overview of the main blood-based biomarker candidates is still lacking. In this narrative review, we discuss the role of proteins, lipids, metabolites, oxidative-stress-related molecules, and cytokines as possible disease biomarkers. Furthermore, we highlight the potential of the emerging miRNAs and long non-coding RNAs (lncRNAs) as diagnostic tools, and we briefly present the role of vitamins and gut-microbiome-related molecules as novel candidates for AD detection and monitoring, thus offering new insights into the diagnosis and progression of this devastating disease.

## 1. Introduction

**Figure 1:**
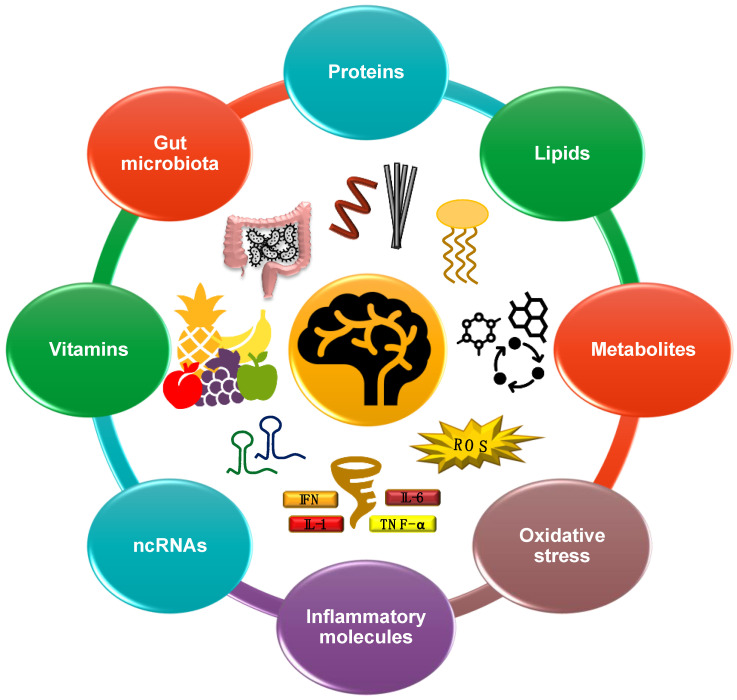
Classification of AD biomarkers. The figure illustrates the classes of blood-based AD biomarkers discussed in this review: long-studied and well-known proteins, inflammatory molecules, lipids, metabolites, oxidative-stress-related molecules, non-coding RNAs, vitamins, and gut-microbiota-based circulating molecules.

Alzheimer’s disease (AD) affects approximately 50,000,000 people worldwide, and is one of the most prevalent and compelling causes of dementia in the geriatric population [1]. Characterized by the extracellular deposition of amyloid-β (Aβ) peptide fibrils and intracellular neurofibrillary tangles, AD has a multifactorial etiology and complex pathogenesis that are still not fully understood [1,2]. To date, no therapy has proved effective against AD, and the high failure rate observed in clinical trials may be due to study design, inclusion criteria, and attempts at treatment when the disease is already at an advanced stage [3,4,5,6]. However, since molecular alterations far precede the onset of neurodegenerative signs, the discovery of new biomarkers associated with early disease stages is of utmost importance [1,2,7]. A biomarker can be defined as a biological marker capable of indicating molecular changes both at a physiological and pathological level [8,9]. An ideal biomarker should be reproducible, highly accurate, non-invasive, cost-effective, easy and quick to measure, and capable of distinguishing between similar conditions without exaggerated technical demand [8,9,10]. Regarding AD, although extensive research has been carried out on Aβ and tau protein alteration in the cerebrospinal fluid (CSF) and via positron emission tomography (PET), high invasiveness and considerable costs remain a concern, thus preventing the implementation of large-scale population screenings [11]. In this respect, the discovery of new minimally invasive blood-based AD biomarkers may be beneficial in presymptomatic diagnosis, disease progression monitoring, drug discovery and development, patient stratification, and targeted therapy [12,13,14,15]. Furthermore, the use of biomarkers to guide preclinical disease stage trials in the context of personalized medicine for neurodegenerative diseases has recently been proposed by the Alzheimer’s Precision Medicine Initiative (APMI), and could represent a breakthrough in AD treatment [13]. Currently, the amyloid-based PrecivityAD™ test is the only recently approved blood test for AD, although phosphorylated tau tests are also promising [7]. However, limitations related to specificity, accuracy, counseling, and interpretation still exist, and solutions based on the combination of several biomarkers belonging to different categories in a single test could strengthen the results [7,16,17,18]. Although extensive research has been conducted, a comprehensive and up-to-date overview of the main emerging blood-based AD biomarker candidates is still lacking. Since several pathways are altered in AD compared to healthy people [1,2], in this narrative review, we analyze the potential of lipids, metabolites, vitamins, inflammatory molecules and cytokines, non-coding RNAs, oxidative stress, and gut-microbiome-derived molecules as possible new blood-based AD biomarkers, thus giving insight into early diagnosis and progression monitoring for this devastating neurodegenerative disease (Figure 1). 

## 2. Methods

To review the potential roles of lipids, metabolites, oxidative stress, inflammatory molecules, ncRNAs, vitamins, gut microbiota, and proteins to function as potential blood biomarkers for AD, we carried out an extensive search in PubMed (U.S. National Library of Medicine) publication database. The following terms were used alone, or in combination, as keywords under the heading “Title/Abstract” to collect and sort our references: “Alzheimer”, “blood”, “serum”, “plasma”, “biomarker/s”, “lipid/s”, “metabolite/s”, “oxidative stress”, “inflammation”, “cytokines”, “inflammatory molecule/s”, “miRNA”, “lncRNA”, “ncRNA”, “vitamin/s”, “microbiota”, “protein/s”. To then systematize the biomarker-category-related literature, the fixed keywords “Alzheimer”, “biomarker/s” and “blood” or “plasma” or ”serum” were combined with each category-related term, according to the 8 sections present in the text. Although recent publications were preferred, our research was not limited by publication date. Finally, book chapters and institutional websites have also been consulted as possible integrative material. 

## 3. Results

### 3.1. Long-Studied and Well-Known Biomarkers: Amyloid-β Peptides and Tau

Several studies indicate the potential for the plasma levels of different amyloid-β (Aβ) variants to function as AD biomarkers, due to their accuracy and predictivity [19,20,21,22,23,24,25,26,27,28,29,30,31,32,33,34,35,36]. This is not surprising considering that CSF Aβ peptides represent one of the core biomarkers in AD diagnosis, and that, at the same time, there is an urgent need to identify more accessible ones [37,38]. On this path, Janelidze et al. suggested that the Aβ_1–42_/Aβ_1–40_ ratio in the plasma could be used as a screening diagnostic marker, followed, when necessary, by more specific tests, such as amyloid PET or CSF Aβ_1–42_/Aβ_1–40_ ratio [39]. This concept is further emphasized by recent discoveries pointing towards a possible association between central nervous system (CNS) Aβ accumulation and increased amounts of several plasma proteins and metabolites (e.g., interleukin 17, α2-macroglobulin, pancreatic polypeptide Y, chemokine ligand 13, vascular cell adhesion protein 1, IgM, apolipoprotein A1, fibrinogen gamma chain, other interleukins, and complement proteins), which may derive from a systemic response to Aβ accumulation [40]. Furthermore, novel, fully automated assays that measure plasma Aβ_1–42_ and Aβ_1–40_ (i.e., Elecsys immunoassays) have been shown to be capable of predicting Aβ pathology in mild cognitive impaired (MCI) as well as AD subjects from both BioFINDER and German biomarker studies, thus encouraging their applications in the context of AD clinical trial prescreenings [41]. 

Another core biomarker suitable for early screening and prognosis is represented by tau, the main constituent of fibrillary tangles, which can be easily detected via PET and in CSF [42,43,44,45]. Nonetheless, again, the invasive nature of CSF biomarkers remains a concern, which prevents them from being used in large-cohort screenings. However, with the recently acquired ability to quantify plasma tau, particularly plasma-tau181 and plasma-tau217, many studies have underscored their feasible use to screen for tau pathology in AD [46,47,48,49]. Of interest, a very recent longitudinal study conducted on elderly subjects enrolled in the Alzheimer’s Disease Neuroimaging Initiative (ADNI) reported that increased plasma levels of p-tau paralleled Aβ pathology in the brain [50], thus suggesting that amyloid plaque deposition is linked to dysregulated tau metabolism, with subsequent release of the soluble p-tau181 in circulation [50]. Of note, data from Karikari et al. demonstrated the high diagnostic accuracy of p-tau181 in identifying AD patients as well as predicting future dementia in a multicenter study conducted on more than 1000 individuals from the ADNI cohort [51]. 

Assessing the ability of blood-based biomarkers to detect the early stages of the disease is pivotal. In this respect, Janelidze et al. conducted an investigation on 176 MCI, 89 subjective cognitive decline (SCD), and 225 healthy individuals from the BioFINDER-2 cohort, and reported that increased levels of plasma of p-tau217 could discriminate preclinical stages of AD prior to any deposition of PET-detectable neurofibrils [52]. Moreover, further evidence from BioFINDER-1 and BioFINDER-2 studies showed that a combination of p-tau217 and plasma Aβ_1–42_/Aβ_1–40_ ratio was able to detect Aβ pathology both in MCI and healthy individuals [53]. 

Overall, these data support the use of plasma p-tau181 and p-tau217 as non-invasive biomarkers for clinical trial recruitment, disease-modifying trial monitoring, prognosis, and diagnosis of any stage of AD progression.

Recent promising results also include DYRK1A kinase, known to be involved in tau phosphorylation and neurofibrillary tangle formation [54]. In this respect, data from the INSIGHT-preAD study reported that plasma DYRK1A levels increase during human aging, but this age-associated rise is blocked in elderly individuals with high brain amyloid load, likely reflecting early brain changes associated with AD during aging [55]. These data point to DYRK1A as a promising theragnostic molecule to be used both as a treatment [54] and as a biomarker to identify people who could benefit from early treatment, as well as for risk stratification [55]. 

### 3.2. Plasma Neurofilament Light

Neurofilament light (NfL) has been listed among the most important AD-associated biomarkers in the Alzbiomarker Database, and NfL levels has been found to increase even at the prodromal stage [56]. However, the fact that high NfL levels are found in association with all neurodegenerative diseases makes this marker less specific to be applied for AD diagnosis [57]. Nevertheless, the recent opportunity of measuring NfL in the plasma through the Simoa assay, in even smaller quantities than before, has made NfL a valuable peripheral biomarker to assess cognitive decline and to identify individuals at risk of neurodegeneration and brain atrophy [58,59]. Accordingly, a recent study by Mattsson et al. on the ADNI cohort, which included volunteers with dementia, MCI, and healthy controls, showed a substantial increase in blood NfL in AD cases compared to controls, and this correlated with disease assessments based on CSF measurements, PET imaging, and cognitive tests [60]. Furthermore, data from Weston et al., comparing familial AD (FAD) mutation carriers and non-carriers, reveal that familial AD patients and presymptomatic carriers both show enhanced circulating NfL compared to non-carriers, with the levels of this protein approximately correlating with the expected time of clinical disease onset [61]. However, given that increased levels of plasma NfL are also found in association with many neurodegenerative disorders [62,63], plasma NfL can be used in the future as a potential screening test to detect neurodegeneration at the primary care unit, while a combination of NfL and other AD biomarkers might be used to monitor disease progression in a clinical trial setting [15,33]. 

### 3.3. Inflammation

#### 3.3.1. Inflammatory Molecules

Inflammation has been considered an important contributor to AD pathogenesis and progression [64,65,66,67,68,69]. Gradual Aβ plaque deposition in the brain and the accumulation of neurofibrillary tangles induce microglia and astrocyte activation, two cell types involved in important physiological roles, such as synaptogenesis, synaptic plasticity, and neuronal support [69]. Although the glial response in physiological conditions is protective, if excessive, it induces a switch from an anti-inflammatory to a pro-inflammatory glial phenotype, thus fostering AD progression [69]. 

Given the central role of inflammation in the development of neurodegenerative diseases, several inflammatory-based fluid biomarkers have been proposed [8,70]. Although most of the data available relate to CSF, many studies have also recently investigated the variation of these molecules in the blood, with promising results [8]. The triggering receptor expressed on myeloid cells 2 (TREM2) is one of the most-studied neuroinflammatory biomarkers [8]. Expressed in microglia, it exerts important physiological functions, such as phagocytosis modulation, cytokine production, and cell division [71]. Recently, increased TREM2 mRNA levels in peripheral blood mononuclear cells (PBMCs) have been found to characterize AD patients compared to controls, and to be dependent on the APOE genotype, in accordance with data obtained using transgenic AD mouse models [72,73,74]. Similarly, evidence from another study of 80 AD patients, 30 amnestic MCI, and 86 healthy volunteers reported enhanced peripheral TREM2 mRNA in AD compared to amnestic MCI, suggesting the ability of this biomarker to discriminate between disease stages [75]. When TREM2 protein expression in circulating monocytes was considered, a test with nearly 70% diagnostic accuracy was obtained by Hu et al., consistent with RNA-based observations [76]. Similar to TREM2, leukocyte mRNA levels of the triggering receptor expressed on myeloid cells 1 (TREM1), which is closely related to TREM2, also follows the same trend, thus representing another possible biomarker [77]. TREM2 levels can also be measured in the form of the so-called soluble TREM2 (sTREM2), the secreted ectodomain of TREM2. Low plasma sTREM2 has been associated with β-amyloid accumulation and CSF p-tau level, but a similar decrease has also been reported in the context of vascular dementia, thus questioning the specificity of this biomarker [71,78]. Alternatively, regarding the plasma levels of soluble TREM1 (sTREM1), opposite results have been reported by Jiang et al., with a gradual rise in this biomarker correlating with AD severity [79]. Although evidence is accumulating, blood-TREM-based biomarkers are still far from clinical application. Indeed, while clear data are emerging on the correlation between CSF sTREM2 and AD, results from a recent meta-analysis have shown there to be no significant difference in plasma sTREM2 levels among AD, MCI, or preclinical AD patients, suggesting that more research is needed to better clarify the role of this biomarker in the blood [80]. 

YKL-40, also known as chitinase-3-like protein 1 (CHI3L1), is a pro-inflammatory glycoprotein expressed in differentiated glial cells, and is considered a marker of neuroinflammation [81,82]. Recently, serum levels of YKL-40 have been shown to be a promising marker for early MCI diagnosis and patient selection, as it is capable of discriminating between cognitive normality and mild cognitive impairment with 85% sensitivity and specificity. However, it was not as good a marker for disease progression [83,84]. Results from a multicentre study have shown that plasma YKL-40 concentration is higher in AD-related dementia, similar to what has been observed with YKL-40 in the CSF [82]. However, it should be noted that high levels of blood YKL-40 have also been reported in aging, vascular dementia, frontotemporal dementia, sporadic Creutzfeldt–Jakob disease, and Lewy body dementia, as well as to vary according to sex, thus almost excluding its applicability as a specific and differential AD biomarker [81,85]. 

Peripheral monocyte chemotactic protein (MCP)-1 and MCP-3 have also been reported to be higher in AD patients than in healthy patients [86], with MCP-1 also being elevated in MCI patients relative to healthy subjects [86,87]. Nevertheless, the statistical significance of the MCP-1 result contrasts with another meta-analysis conducted by Olsson et al. in 2016 [56]. Interestingly, Morgan et al. recently showed that a panel of ten proteins, including cytokines eotaxin-1, MCP-1, and MIP-1β, was able to significantly differentiate AD, MCI, and healthy control groups, with subsets of this panel also successful in discerning patients from controls when tested in a discovery cohort [70]. 

C-reactive protein (CRP) is an acute-phase protein, the level of which rises during inflammation; however, evidence for its use as an AD biomarker remains inconclusive. Indeed, while some studies indicate that blood CRP levels correlate with Mini-Mental State Examination (MMSE) score, other results suggest that this is valid only among APOEε4 homozygote AD patients [88,89]. Still, no association with cognitive decline has been reported from independent data, leaving CRP as a debatable biomarker [90,91]. 

Chemokines are a set of chemo-attractant cytokines that participate in the inflammatory process, and are involved in dementia development [92,93]. In this respect, while blood CX3CL1, also called fractalkine, has been found to be upregulated both in MCI and AD, plasma CCL23 seems to better predict MCI-to-AD progression [93,94]. Plasma concentrations of the C-C chemokine ligand (or RANTES) have been reported to be elevated in AD and to correlate with an increased inflammatory burden [95,96,97]. However, since changes in RANTES have also been noted in other neurodegenerative and metabolic diseases, further research is needed to better understand the specificity of this marker [95,98,99]. 

The complement system plays a key role in innate immune defence, and a strong inflammatory response is produced upon its activation [100]. In this respect, while increased circulating clusterin (a member of the small heat shock protein family also involved in complement-mediated cell lysis) has been measured in AD compared to controls, a combination of clusterin, factor I, and terminal complement complex evaluation can discriminate between MCI subjects that will develop dementia and those who will remain stable [101,102]. On the contrary, significantly lower levels of complement component 3 (C3) have been reported in the serum of AD patients compared to healthy volunteers, but no correlation was found when complement component 4 (C4) was considered [89]. 

Other inflammatory proteins have also been hypothesized as possible AD biomarkers, but data remain uncertain. For example, Suidan et al. found that young AD patients are characterized by delayed clotting [103]. However, since the clotting profile may change considerably in many different conditions, additional data on larger cohorts are warranted [103]. Similarly, interferon-γ-induced protein 10 (IP-10), an important player in inflammation and angiogenesis [104], has been reported to be the plasma analyte from the ADNI cohort showing the highest abnormality levels [105]. However, no correlation between serum levels of IP-10 and AD or MCI was found in an independent study by Galimberti et al. [106], although positive results had previously been described concerning CSF IP-10 content [107]. Regarding immunoglobulins, while serum IgA and IgG levels have been shown to be significantly higher in AD compared to controls, no differences were found for IgM [89]. Finally, since a single inflammatory molecule might often lack specificity, and is subject to interpersonal variations, a combination of possible biomarkers might represent an option to strengthen predictive capacity, as already reported [108,109,110].

#### 3.3.2. Circulating Cytokines 

Dysregulation of inflammatory cytokines has been shown in the brain tissue of AD patients in post mortem analyses as well as in the CSF of MCI and AD patients [56,111]. These results led to increasing interest in the role of circulating cytokines in AD, since they may circumvent the need for invasive diagnostic procedures.

A sizable body of evidence has been accumulated on how blood cytokine levels differ across AD, MCI, and non-demented patients, although disagreements over the conclusions persist. In their meta-analysis, Bradburn et al. established blood IL-6 as a risk factor for cognitive decline in MCI patients, with high IL-6 levels being associated with an increased risk of an AD diagnosis at a follow-up visit within 2–7 years (odds ratio, 1.42) [112]. La Rosa et al., following MCI patients for 2 years, found that blood samples collected at baseline had higher PBMC mRNA levels of IL-1β and IL-6 in AD converters than in non-converters only if the samples were stimulated with Aβ, suggesting that an inflammatory *milieu* may contribute specifically to the onset of AD [113]. Elevation in pro-inflammatory cytokines, such as interleukin (IL)-6, tumor necrosis factor (TNF)-α, IL-1β, transforming growth factor (TGF)-β, IL-12, and IL-18, in the peripheral blood of AD patients compared to control subjects was also reported by Swardfager et al. in a meta-analysis comprising 44 studies [114]. Moreover, in a more recent study by Lai et al., in addition to the previous markers, circulating IL-2, interferon (IFN)-γ, CRP, and CXCL10 were found to be elevated in AD patients compared to healthy controls, while IL-6 levels were inversely correlated with cognitive function, in contrast with other studies [115]. Some of these findings were confirmed in another meta-analysis, which also found that soluble TNF receptor (sTNFR)-1 and sTNFR-2 are overexpressed in the blood of AD patients relative to healthy controls or MCI patients [87]. However, yet another review on peripheral IL-1β, IL-6, TNF-α, and CRP found no statistically significant difference between AD patients and controls in any of these markers [116]. A possible explanation for this discrepancy is that the meta-analysis by Ng et al. included far fewer studies than that by Lai et al. Still, Nesham et al. reported the opposite trend in a study of 60 subjects, in which mRNA levels of IFN-γ and TNF-α in PBMCs of AD subjects were, instead, decreased compared with non-demented controls [117]. 

Studies have also investigated whether other circulating cytokines can predict the conversion of MCI to AD. One study showed that the absence of IL-33 was more common in MCI patients who converted to AD at 1-year follow-up visits than in those who did not convert to AD; overall, the cognitive function in patients expressing IL-33 was better preserved than in patients who did not express it [118]. Similarly, one report suggested that osteopontin (a matricellular protein originally isolated from bone, also functioning as a pro-inflammatory cytokine) is more highly expressed in the blood of recently diagnosed AD patients than in those that have had AD for more than 2 years [119]. Furthermore, it was also found that MCI patients had higher blood osteopontin levels at diagnosis of AD progression [119]. Moreover, within a panel consisting of 29 cytokines, and including total tau protein, p-tau181, Aβ_1–40_, and Aβ_1–42_, a high level of circulating IL-2 was found to be the best-performing biomarker to predict a slower cognitive decline in MCI patients (measured by a two-point decrease, or more, in the MMSE), though no reliable biomarker was found in AD patients [120].

### 3.4. Metabolism

Metabolites are defined as the intermediate and final products of metabolic reactions. Usually, this term is used to indicate relatively small biomolecules involved in various biological processes, such as cell growth, reproduction, food breakdown, and chemical detoxification, and they constitute the building blocks of many other biological components [121]. Disruptions to many biochemical pathways, such as amyloid precursor protein metabolism, tau protein phosphorylation, oxidative stress, mitochondrial function, inflammation, lipid metabolism, and neurotransmitter pathways, occur in AD patients [122]. Therefore, metabolomics analysis (MA) may represent a new method to investigate a multifactorial disease, such as AD, because of its ability to detect hundreds of metabolites rapidly and synchronously [123,124].

Furthermore, research has highlighted that the biochemical mechanisms underlying AD start decades before the clinical onset of dementia [125], which offers the opportunity to use biomarkers as an adjunctive tool for early AD diagnosis [126]. According to these discoveries, biomarkers can be added into the diagnostic procedure to recognize specific phases of disease progression, to assist doctors in monitoring the course of AD, and to improve the accuracy of the diagnosis [127,128]. 

A recent study conducted by Sun et al. on 30 AD patients, 32 MCI patients, and 40 controls found 11 metabolites able to discern between AD patients and controls [129]. In particular, 1,4-butanediamine and L-ornithine, compared to the other metabolites, turned out to have a higher diagnostic capacity [129]. Outcomes of this study suggest that irregular energy metabolism, oxidative stress, and metabolic disorders of lipids and amino acids in patients affected by AD or anamnestic mild cognitive impairment (aMCI) might occur [129]. Furthermore, recent evidence by Piubelli et al. suggests that both serum D serine concentrations and D-/total serine levels are indicative of disease progression, and can represent new advanced biomarkers [130].

Several studies indicate that AD is highly prevalent in adults with Down syndrome (DS), and, therefore, biomarker discovery in this population is of interest [131,132]. In this context, Gross et al. examined plasma samples from 78 patients with Down syndrome who met the diagnostic criteria for AD (DS-AD) and 68 individuals with Down syndrome who did not (DS-NAD) [133]. Outcomes revealed remarkably higher levels of lactic, pyruvic, and methyladipic acids in the DS-AD group in comparison to the DS-NAD group, suggesting that, in this population, AD is accompanied by a switch from aerobic respiration to fermentative, less efficient metabolism [133]. In addition, markedly decreased levels of uridine were noticed in the DS-AD group, without evidence of hypoxia [133]. However, since all participants were affected by Down syndrome, the absence of healthy controls could represent a possible limitation to this study given the similar pathological aspects and cellular dysfunctions in DS and AD [131,134]. Of note, dysfunctions in mitochondrial bioenergetics accompanied by a shift in glucose metabolism have also been reported to long precede the onset of neurotypical AD, suggesting that the observations regarding the DS-AD population may be applicable even in AD patients without other comorbidities [135,136,137,138].

Significantly increased plasma levels of lithocholic acid (LCA) have been detected in AD patients in comparison to healthy controls [139]. The same study, in which the levels of 20 bile acid metabolites were quantified in plasma, also reported higher levels of glycochenodeoxycholic acid (GCDCA), glycodeoxycholic acid (GDCA), and glycolithocholic acid (GLCA) in AD compared to MCI patients. However, although LCA and GDCA may be useful to routinely diagnose AD using plasma samples, this analysis revealed a limited specificity, sensitivity, and accuracy compared to other plasma markers. Thus, these two bile acid markers measured in the plasma could be helpful to diagnose AD in combination with other biomarkers [139].

Associations between circulating metabolites and neocortical amyloid positivity were also investigated [140]. In this regard, a panel consisting of anandamide and its isotope, phosphatidylethanolamine, phosphatidylcholine, and an unidentified metabolite with a median mass/charge ratio of 829.66, was found to be able to predict PET neocortical amyloid burden with 72% accuracy, with the potential to develop a simple blood test to diagnose AD even at the prodromal or preclinical stages [9,140]. Although a promising candidate for monitoring the progression of amyloid pathology in anti-amyloid trials, the ability of this signature to differentiate demented from non-demented individuals remains to be validated, as there are subjects who show the amyloid signature neuropathologically, yet are cognitively intact [141,142,143]. 

Results concerning the Alcadeins (Alcs) family are also of interest [144]. Given that the quantity of p3-Alcα in plasma mirrors the pathological process of Aβ build-up in AD patients, it was hypothesized that the quantity of p3-Alcα could be used as a plasma biomarker in AD [145]. Indeed, it has been reported that plasma p3-Alcα concentrations in AD and MCI patients were significantly enhanced in comparison with controls. Elevated p3-Alcα plasma levels turned out to be remarkably correlated with AD risk, despite adjustment for confounding factors, including age, gender, ApoE-ε4, and renal function [145]. 

Acylcarnitines are a large class of metabolites that play key roles in long-chain and branched-chain fatty acid metabolism, insulin resistance, cellular stress responses, and cholinergic neurotransmission [146,147,148,149]. Of interest, three of these acylcarnitines, decanoylcarnitine [C10], pimelylcarnitine [C7–DC], and tetradecadienylcarnitine [C14:2], were predictive of a lower risk of AD onset [150]. However, a possible weakness of this study may be the fact that lower plasma concentrations of decanoylcarnitine and tetradecadienylcarnitine were also found in individuals with schizophrenia compared with healthy controls, thus limiting the specificity of these biomarkers [151]. Following this analysis, several other studies have designed interesting diagnostic panels. A cross-sectional study found several metabolites, the levels of which were altered both in AD patients and MCI patients [18]. All these data were used to create a logistic regression model that precisely discriminates AD from normal controls [18]. Seven metabolites composed the final panel: one non-esterified fatty acid (22:6n − 3, DHA), one bile acid (deoxycholic acid), one sphingomyelin (SM(39:1)), three amino acids (glutamic acid, alanine, and aspartic acid), and one phosphatidylethanolamine (PE(36:4)). This metabolic signature was even able to distinguish between MCI and normal control patients, suggesting that it may be a powerful resource for early-stage diagnosis [18]. Another biomarker panel consisting of six plasma metabolites belonging to amino acid metabolism, one-carbon metabolism, and fatty acid and nucleic acid metabolism (arachidonic acid, N,N-dimethylglycine, thymine, glutamine, glutamic acid, and cytidine) was able to discriminate AD patients from normal controls [152]. Overall, these results supply a broad global plasma metabolite profile, and may strengthen early diagnosis [153]. 

Finally, data from a combined omics analysis performed on the INSIGHT-preAD cohort showed that a combination of metabolomic and transcriptomic features was able to discriminate between amyloid-negative and amyloid-positive individuals, with the potential to be applied in early screenings and in risk stratification assessments [154].

In conclusion, blood MA provides hope for a better comprehension of AD, as well as for early diagnosis and prompt therapy, but more research is required to address its specificity and reproducibility [151,155].

### 3.5. Oxidative Stress

Oxidative stress is characterized by the loss of balance between reactive oxygen species (ROS) and antioxidant defenses, leading to protein and DNA oxidation, lipid peroxidation, glycoxidation, and altered glucose metabolism [156]. Since the brain has a high rate of oxygen consumption, is constituted by lipids that can be easily oxidized, and contains less antioxidant molecules than other organs, it is considered particularly exposed to oxidative damage [157]. Several studies have reported that oxidative stress can cause early brain alterations, and thus hypothesized a central role of oxidative damage in the pathogenesis of many neurodegenerative diseases, including AD [158,159,160]. Up to now, lipid peroxidation and many oxidative-stress-related molecules have been detected as differentially expressed in AD brain, urine, and/or CSF compared to controls, such as 3-nitrotyrosine, 4-hydroxynonenal, and 8-hydroxy-2-deoxyguanosine [161,162]. Recently, blood has also been considered a source for oxidative-stress-based biomarkers, and Table 1 summarizes the main findings [10,163,164,165,166,167,168,169,170,171,172,173,174,175,176].

Glutathione (GSH), a tripeptide composed of cysteine, glutamate, and glycine, represents the most abundant and dominant endogenous antioxidant in the body; its homeostasis has been reported to be dysregulated in neurodegenerative diseases [177]. Regarding AD, while serum GSH levels are significantly lower in patients than controls, its plasma concentration has been correlated with cognitive decline, and it is able to discriminate between MCI subjects and healthy volunteers [163,164]. Despite these data, L-cysteine prodrug supplementation, or oral γ-glutamylcysteine administration, did not prevent AD alterations, nor did it restore GSH and oxidative markers, thus underscoring the need for further studies [178]. Similarly, serum glucose-6-phosphate dehydrogenase (G6PD), an enzyme that protects red blood cells from oxidative stress, has been found to nearly double in AD subjects relative to healthy individuals, although more studies are required to strengthen these results [165]. Plasma levels and the activity of other enzymatic antioxidants, such as extracellular superoxide dismutase, catalase, and glutathione peroxidase, were shown to decrease progressively according to the severity of the cognitive impairment [169,176]. Likewise, when Zengi et al. compared 21 moderate AD subjects with 20 healthy volunteers, significantly low blood levels of the high-density lipoprotein-associated antioxidant enzyme paraoxonase 1 have been detected in the AD group, thus confirming ROS/antioxidant imbalance as an AD signature [168]. Alternatively, among mild cognitive impaired type 2 diabetic patients, enhanced plasma activity of the enzyme dipeptidyl peptidase-4 (DPP4), known to enhance inflammation and oxidative stress, is negatively linked with circulating brain-derived neurotrophic factor (BDNF) levels, and positively correlates with inflammatory markers (i.e., IL-6, CRP) and cognitive impairment [179,180].

Isoprostanoids are the result of non-enzymatic oxidation of polyunsaturated fatty acids, and are the secondary product of lipid peroxidation [181]. However, while some evidence suggests increased plasma dihomo-isoprostanes and neuroprostanes in AD patients, other studies show no significant difference from controls [166,182].

Several minerals and vitamins are also known to exert an antioxidant effect, although evidence for their potential use as AD biomarkers remains debated [183]. Indeed, while plasma and erythrocyte selenium concentrations have been reported to decrease in cognitively impaired individuals, other evidence shows that serum levels of the same mineral seem not to change in overt AD [170,184]. Similarly, alterations in iron, zinc, and copper have also been described [185,186,187,188]. For instance, Mueller et al. reported that an increased serum copper/non-heme iron ratio can predict the progression from mild cognitive impairment to overt dementia, thus representing a promising early diagnostic biomarker [187]. However, results from another study conducted on 36 AD patients, 18 MCI individuals, and 33 controls did not find copper to have the capacity to differentially diagnose AD and MCI conditions, thus calling for new studies [176].

Other potential markers directly or indirectly associated with oxidative stress imbalance, such as sialic acid deficiency, increased protein carbonylation, plasma unfolded p53, acetylcholinesterase (AChE) expression, serum thiol–disulfide balance, and serum ischemia-modified albumin (IMA) concentration, have been investigated in independent studies, but their results need to be replicated [10,172,189,190,191]. Interestingly, when 113 patients with aMCI were compared to 832 controls, serum IMA amount, and the IMA/albumin ratio, were shown to be capable of detecting AD at the prodromal stage, suggesting the potential for this molecule to detect early disease onset [172].

Other possible oxidative-stress-related biomarkers have also been investigated. For example, serum uric acid levels have been found elevated in the preclinical stage of AD, and this increase was particularly pronounced in people with amyloid pathology [173]. Since some studies indicate that hyperuricemia may act as an antioxidant [192], these results suggest that increased uric acid levels may represent an antioxidant response of the body against amyloid load, which is not only present at the preclinical AD stage, but also characterizes the clinical stage. Recently, results from another cross-sectional study carried out on a total of 496 individuals show that low plasma ergothioneine levels, an uncommon sulfur-containing derivative of the amino acid histidine with antioxidant properties, typify demented patients and inversely correlate with disease severity, although it could not distinguish between AD and vascular dementia [171]. Moreover, altered levels of redox-reactive antiphospholipid antibodies (directed against the plasma protein β2-glycoprotein I and not phospholipids), decreased serum concentrations of some sirtuins (SIRT1, SIRT3, and SIRT6), and reduced serum coenzyme Q10 concentrations have also been considered as disease markers, although, regarding coenzyme Q10, discordant data have been published [174,175,193,194].

Finally, erythrocyte morphology, membrane protein composition, and oxidative stress hallmarks have been proposed as possible circulating biomarkers, but more research is needed [195].

Overall, when considering the potential use of oxidative-stress-related molecules as possible biomarkers, it should be noted that oxidative damage is a common hallmark of all neurodegenerative diseases, and is found in several other conditions, thus making it difficult to find a specific AD marker [161]. Nevertheless, the need for non-invasive disease biomarkers coupled with these promising emerging data should encourage new large, comprehensive, and confirmatory studies to be undertaken.

### 3.6. Circulating Non-Coding RNAs

#### 3.6.1. miRNAs—Alzheimer’s Disease

MicroRNAs (miRNAs) are short non-coding RNAs (approximately 21 bp long), with an important role in post-transcriptional gene modulation [196,197,198]. They can circulate in the blood, either as cell-free miRNAs bound to specific proteins or encapsulated in microvesicles, typically exosomes [199]. RNA sequencing and next-generation sequencing (NGS), microarray analysis, and quantitative reverse transcription–polymerase chain reaction (RT–PCR) are all suitable techniques typically used to detect miRNA levels in the bloodstream [199,200]. Dysregulations in blood and CSF miRNA levels have been reported during aging and in age-related diseases, including neurodegeneration [200,201,202,203,204,205,206,207]. This aspect, in association with the fact that they are stable in biofluids, makes miRNAs ideal non-invasive biomarkers for early diagnosis, disease progression monitoring, population screenings, and even therapy [199,200,208,209]. Regarding AD, early evidence came from Kumar et al., who reported that a miRNA signature consisting of seven plasma miRNAs (hsa-let-7d-5p, hsa-let-7g-5p, hsa-miR-15b-5p, hsa-miR-142-3p, hsa-miR-191-5p, hsa-miR-301a-3p, and hsa-miR-545-3p) can differentiate AD and healthy individuals with more than 95% accuracy [210]. Soon after, Kiko et al. proposed that the decrease in plasma miR-34a and miR-146a found in AD patients compared to controls could be used to non-invasively detect the disease [211]. Subsequent investigations have shown that upregulation of miR-590-5p and miR-142-5p, along with downregulation of miR-194-5p, is distinctive of AD subjects compared to healthy volunteers [212]. Moreover, enhanced plasma concentrations of hsa-let7d-5p and hsa-let7g-5p were detected in a study comprising 50 AD and 50 age- and gender-matched controls [213].

PBMCs have also been considered a source of potential miRNA-based biomarkers [214]. For instance, decreased hsa-miR-29b in PBMCs has been correlated with lower SP1 expression, a transcription factor that regulates the transcription and translation of proteins involved in AD [215]. Although both hsa-miR-29b and hsa-miR-375 are closely related to SP1 regulation, the results are not significant [215]. Instead, data derived from an array analysis carried out on PBMCs from 16 AD patients and 16 gender-, age-, and ethnicity-matched controls showed significant upregulation of miR-34a and miR-181b in demented subjects, while the expression of several other miRNAs changed according to APOE genotype [214].

As far as pathophysiological modifications are concerned, miRNAs have been reported to modulate Aβ levels through the regulation of the amyloid precursor protein (APP) and tau phosphorylation, as described for miR-455-3p and miR-483-5p, respectively [216,217]. In this context, plasma levels of miR-2000-3p, a neuroprotective ncRNA against Aβ-mediated toxicity, were reported to be lower in AD compared to controls, both in vitro and in vivo [218]. Moreover, levels of circulating miR-15a have been shown to correlate with plaque score [219]. In parallel, Geekiyanage et al. proposed four downregulated serum miRNAs involved in Aβ and tau phosphorylation pathways (miR-137, miR-181c, miR-9, and miR-29a/b) as a potential panel for early disease screenings [220]. However, while further investigations conducted on 105 AD and 150 healthy individuals led to the validation of Aβ-regulated miR-181c as being decreased in the serum of AD patients compared to controls, contrasting evidence was found for miR-9, with increased levels of this biomarker also reported in demented individuals [72]. BACE1, SP1, NCSTN, PTEN, and SIRT1 are also regulated by miRNAs implicated in AD, with miR-9, miR-16, miR-34a, miR-106a, miR-107, miR-125b, miR146, and miR-181c presenting the highest level of interactions in the network [221]. In support of this, consistent decreases in circulating miR-29c levels, a miRNA negatively correlated to BACE1 expression, were found to characterize AD patients compared to age-matched controls [222]. Moreover, the downregulation of three miRNAs that modulate target proteins related to AD, such as APP and CaMKK2 (hsa-miR-9-5p, hsa-miR-106a-5p, and hsa-miR-106b-5p), have been correlated with disease severity; hsa-miR-106a-5p alone reaching statistic values of 93% specificity and 68% sensitivity in AD diagnosis [223]. Other possible biomarkers have been identified by Liu et al. in a study comprising 50 AD patients, 20 individuals with vascular dementia (VD), and 50 healthy controls, in which a significant decrease in the circular RNA hsa-circ-0003391 in peripheral blood was correlated with a rise in miR-574-5p in AD compared to both controls and VD subjects [224]. When machine learning approaches were applied to a total of 465 subjects, including AD and controls, circulating levels of miR-532-5p showed the highest correlation with neurodegeneration (AUC 87,6%), but miR-26a/26b-5p were the best predictors of MMSE score [225].

Since immune modulation is of central importance in AD pathophysiology, the circulating miRNAs involved in these pathways could represent a source of novel early AD biomarkers. In this respect, an increase in circulating miR-206 levels in AD patients has been found to correlate with enhanced inflammation and reduced expression of the neuroprotective factor IGF1 [226]. In addition, miR-146b-5p and miR-15b-5p downregulation, two miRNAs involved in innate immune system regulation and cell cycle control, have been linked to AD after performing RNA sequencing on 40 amyloid-positive AD patients and 31 amyloid-negative healthy controls [227]. Given the large amount of data usually generated when comparing the expression of hundreds of miRNAs in large cohorts, recently, machine learning approaches have also been considered and miRNA-based biosignatures have been proposed [228]. When random forest-based machine learning approaches were used to account for miRNA dysregulation, brain volume, comorbidities, and demography, three blood miRNAs related to cellular senescence and inflammation were found to be the best predictors of cognitive impairment: miR-140-5p, miR-197-3p, and miR-501-3p [229]. Moreover, a deregulation in blood miRNAs involved in neuroinflammatory pathways has been reported by Yuen et al. in based on their results from a meta-analysis followed by machine learning techniques [230]. To date, serum levels of miR-125 are among the most promising ncRNA-based biomarkers [72]. In this respect, upon analyzing 84 AD and 62 healthy subjects, Jia et al. reported a significant decline in serum miR-125b and miR-223, both involved in immune regulation, with a combination of both being more predictive than either miRNA alone [231]. Interestingly, serum miR-125b levels have also been reported to be capable of discriminating AD patients from both healthy controls and subjects characterized by inflammation, thus excluding neuroinflammation as a possible confounding effect [232]. Finally, levels of the closely related miR-34c, implicated in repressing cell survival and antioxidant defense, were found to be upregulated in plasma from AD individuals compared to age-matched controls, in an independent study [233].

Diminished concentrations of other ncRNAs, such as hsa-miR-501-3p, were reported to correlate with MMSE score, while an opposite trend has been observed for miR-455-3p [234,235]. Notably, contrasting results have been obtained when analyzing the levels of the DNA replication modulator hsa-miR-501-3p in the brain of AD subjects, suggesting an intermittent concordant trend between serum and other compartments [234].

Despite promising results, reproducibility and validating issues often pose problems in identifying one or several miRNAs that can be used in disease diagnosis. Therefore, using a group of biomarkers might represent a strategy to strengthen the results and decrease interindividual variability. For example, after performing NGS on blood samples from 49 AD, 20 MCI, 90 multiple sclerosis (MS) patients, and 55 controls, Keller et al. proposed a pool of 68 miRNAs as an AD diagnostic set [236]. In addition, when conducting a genome-wide serum microRNA screening with NGS, and subsequent RT–PCR, on a discovery cohort and a validation cohort, six miRNAs were differentially expressed between patients and controls (miR-98-5p, miR-885-5p, miR-483-3p, miR-342-3p, miR-191-5p, and miR-let-7d-5p), with miR-342-3p presenting the highest sensitivity and specificity [237]. Results from an integrated analysis conducted on 12 miRNA datasets identified 37 dysregulated miRNAs in AD compared to controls, with has-miR-93, has-miR-26b, has-miR-34a, has-miR-98-5p, and has-miR-15b-5p being the key nodes when analyzing miRNA–mRNA interactions and modulation [238]. Notably, machine learning techniques can also be applied to analyze peripheral blood miRNA signatures, with recent data proposing machine learning models reaching up to 92% and 90,9% accuracy in the serum and plasma, respectively [239]. Lastly, a combination of 12 miRNAs has been shown to be able to differentiate AD and controls with an accuracy of 93% and a specificity of 95%, thus clearly improving the statistical strength [17]. However, predictive values of the same signature decreased to 74–78% accuracy when used to distinguish between AD, MCI, Parkinson’s disease (PD), depression, bipolar disorder, and schizophrenia [17].

Regarding blood-based miRNA platforms dedicated to differential diagnosis, results from a multicenter study showed that serum is even better than CSF to discriminate and classify patients with the sporadic behavioral variant of FTD from AD, with upregulated miR-223-3p and downregulated miR-15a-5p seeming to characterize the former [240,241]. Furthermore, AD and VD might be differentially diagnosed by measuring miR-31, miR-93, and miR-146a serum concentrations [242], while other miRNAs can be used alone or in combination to distinguish between AD, ALS, and controls [241]. A miRNA signature based on 37 brain-enriched and plasma miRNAs also proved able to distinguish between AD, PD, frontotemporal dementia (FTD), and amyotrophic lateral sclerosis (ALS), albeit with varying levels of accuracy [243]. In addition, results from a study performed on 120 AD, 120 PD, and 120 healthy controls revealed that, while both plasma miR-103 and miR-107 are lower in AD compared to controls, and are both correlated with MMSE score, only miRNA-103 is capable of significantly differentiating AD from PD [244].

Overall, miR-125b seems to be one of the best-characterized circulating AD ncRNAs, with recent evidence supporting its use as a theragnostic biomarker [72,245]. More research is needed to better clarify the role of other potential ncRNAs in early disease detection.

Table 2 relates the main findings on circulatory ncRNA dysregulation in AD [17,210,211,212,213,218,220,222,223,224,225,226,227,231,232,233,234,235,237,238,242,246,247,248,249,250,251,252,253,254,255,256,257,258,259].

#### 3.6.2. miRNAs—Early Diagnosis

Since early diagnosis in AD is of utmost importance, and different AD stages (mild, moderate, and severe) are reported to be characterized by a distinct set of dysregulated serum miRNAs, many studies have been carried out to differentiate early disease onset from late AD stage and/or healthy controls (Table 2) [206]. Regarding MCI, while reduced serum miR-31, miR-143, miR-93, and miR-146a have been found capable of discriminating between AD and controls, the last two were also shown to be upregulated in MCI versus healthy individuals [242]. Moreover, Sheinerman et al. reported that two plasma miRNA families, miR-132 and miR-134, are both independently capable of distinguishing between MCI and aged-matched controls, with no gender differences [260,261]. Two pairs of plasma miRNAs (hsa-miR-191, hsa-miR-101 and has-miR-103, has-miR-222) have also been shown to have high accuracy for detecting MCI [262]. Of note, enhanced serum concentration of miR-34c was correlated with the aMCI stage, similar to that observed in the plasma of AD patients [233,253]. Concerning the potentiality of using ncRNA to monitor disease onset and progression, while diminished levels of serum miR-384 and miR-210 have been reported to correlate with disease severity [247,256], plasma levels of miR-342-5p inversely correlated with cognitive manifestations at a 2-year follow-up in another study [252]. Furthermore, of the plasma miR-15b-5p, miR-142-3p, miR-34a-5p, and miR-545-3p reported to distinguish AD from controls, only the last two could discriminate preclinical AD from AD and healthy individuals. However, the lack of corroboration regarding these data in the validation cohort underlines the need for deeper investigation [249].

Combinations of other plasma biomarkers have also been proposed. Nagaraj et al. reported that, among 15 miRNAs prioritized from a wider screening, six plasma miRNAs were able to detect AD at an early stage compared to healthy individuals [248]. A prodromal AD biosignature consisting of five plasma miRNAs (miR-1185-2-3p, miR-1909-3p, miR-22-5p, miR-134-3p, and miR-107) was instead proposed by He et al. based on microarray sequencing performed on three different datasets (discovery, analysis, and validation cohorts) [250]. Finally, a combinatorial signature comprising diet, gut microbiota, and serum miRNA biomarkers has also been reported to distinguish between MCI and controls, suggesting that joining different biomarkers in the same diagnostic test could reduce unpredictable variabilities [254].

Despite the promising results, clearly differentiating MCI from AD remains a challenge. Indeed, even though plasma miR-92a-3p, miR-181c-5p, and miR-210-3p were all reported to be higher in MCI than AD, they are significantly increased in both MCI and AD when compared to controls [251]. Similarly, although plasma miR-483-5p was found to be elevated in MCI compared to AD, both conditions presented a significant rise in this miRNA in plasma compared to healthy individuals, thus making it difficult to distinguish between the two stages [246].

#### 3.6.3. miRNAs—Exosomes

Exosomes are highly stable small membrane-enclosed vesicles (diameter of 30–100 nm) originating from the cellular biosynthetic secretory pathway, and are used to transport RNAs, proteins, and lipids in circulation [263,264,265]. Being able to resist the activity of ribonucleases, they offer protection to their cargoes, thus representing a source of biomarkers less susceptible to interference compared to cell-free blood miRNAs [263]. Due to these characteristics, CNS-derived blood exosomes have already been considered a diagnostic tool in different neurodegenerative diseases, such as AD, PD, and ALS [263,264,265,266]. In AD, both plasma- and serum-derived exosomes have been investigated as a source of novel AD biomarkers, sometimes obtaining discordant results compared with the respective cell-free miRNA levels [259]. Concerning plasma, in 2015, Lugli et al. reported a panel of 21 exosomal-derived miRNAs differentially expressed between AD and controls, among which seven were highly discriminating, reaching an accuracy of 83–85% for AD detection [267]. Moreover, while downregulation of miR-212 and miR-132-3p in plasma extracellular vesicles was reported to discriminate between AD and controls, the latter was not found capable of differentiating MCI from healthy individuals [268]. Diminished levels of circulating ex-miR-342-3p, ex-miR-125a-5p, ex-miR-125b-5p, and ex-miR-451a were also detected in AD patients [269]. However, it should be noted that a similar deregulation in exosomal miRNAs has also been reported during normal aging, thus evidencing the importance of age-matched controls [269]. Results from another exploratory study conducted in 2019 showed that reduced levels of hsa-miR-23a-3p, hsa-miR-126-3p, hsa-let-7i-5p, and hsa-miR-151a-3p could efficiently distinguish AD from healthy subjects, while hsa-miR-451a and hsa-miR-21-5p performed better in differentially diagnosing between AD and Lewy body dementia [270]. Regarding other pathologies, different signatures were also established from large and small extracellular vesicle-derived miRNAs to distinguish between AD, PD, ALS, and FTD, thus underscoring the potentiality of these biomarkers [271].

Concerning serum, there is evidence of machine learning methods, based on random forests and accounting for neuroimaging and clinical data, that are capable of predicting AD upon defining the optimal miRNA signature [272]. In addition, while ex-miR-223 was found to be downregulated in AD, upregulation of ex-miR-135a was related to both MCI and AD in a study comprising 131 MCI, 198 AD, and 30 healthy controls [273,274]. However, other results show that, while rising serum ex-miR-135a is a feature of AD patients, a combination of ex-miR135a, ex-miR-384, and ex-miR-193b seems to better define MCI individuals, thus evidencing the need for further analysis [275].

#### 3.6.4. miRNAs—Limitations

Although miRNAs present real promise for future biomarker discovery, several limitations remain to be addressed. First, although many miRNAs are significantly dysregulated, the lack of reproducibility between studies prevents consistency, with only one or a few miRNAs being replicated by independent groups [276]. In addition, gender and age differences should also be considered, as some miRNA levels vary in a sex-dependent manner [243]. Secondly, since dysregulations in similar miRNAs, such as miR-29, miR-26, and let-7, have also been reported in PD [277], and a plethora of ncRNAs have been implicated in the pathogenesis of several neurodegenerative diseases [278], careful analysis of their ability to differentially diagnose these conditions is required. Concerning technologies, it should also be emphasized that high throughput methods can analyze hundreds of miRNAs at the same time, but can have low sensitivity and high variability depending on the extraction method and on the normalization applied [208]. In this respect, the analysis of single nucleotide polymorphism variation in the miRNA biogenesis pathway and microfluidic-based quantitative PCR platforms has recently been proposed to partially solve these issues [279,280].

Regarding exosomes, although the methods used for their isolation and purification are expected to be reproducible, several procedural differences among distinct laboratories need to be revised to reach a consensus protocol and reduce background noise [281]. Overall, standardized measurement techniques, reproducible and universal protocols for sample collection and purification, large cohorts, clear criteria for the classification and design of the study, and standard statistical analysis with defined cut-offs are of utmost importance, and should be considered when designing new investigations [265,276].

#### 3.6.5. Long Non-Coding RNAs

Long non-coding RNAs are a subset of non-coding RNAs (ncRNAs) characterized by long transcripts (>200 nucleotides) devoid of protein-coding function [282]. They have been implicated in the regulation of several biological processes, such as proliferation, transcriptional and post-transcriptional regulation, malignancies, and apoptosis [282,283,284]. Concerning AD, recent insights have shown the involvement of ncRNAs and lncRNAs in disease pathogenesis, therefore creating interest around the possibility of their use as biomarkers (Table 2) [285,286,287]. Although there is currently less evidence than for miRNAs, altered brain expression of the RNA polymerase III-dependent ncRNA (i.e., NDM29, of the β-site amyloid precursor protein cleaving enzyme-1-antisense lncRNA (i.e., BACE1-AS), and of the intronic ncRNA (i.e., S1A) is known to be correlated to Aβ formation [287,288,289]. Moreover, increased cerebral tissue transcription of two lncRNAs, linc00507 and 17A, has been implicated in tau phosphorylation and GABA B alternative splicing, respectively [290,291,292]. Concerning possible circulating biomarkers, Feng et al. report evidence for significant plasma lncRNA BACE1 upregulation in 88 AD patients when compared to 72 controls [257]. However, no significant differences were found regarding plasma lncRNA 17A, S1A, or BC200, despite previous evidence showing alterations in some of these lncRNAs in the brain [257,289,291]. These data were also confirmed in 2020 by Wang et al., who reported a consistent rise in plasma exosomal lncRNA BACE1-AS in a study on 72 AD and 62 healthy individuals, reaching 87.5% sensitivity and 61.3% specificity [293]. However, another study comparing 45 AD and 36 control subjects reported low BACE1-AS in the pre-AD stage, while these levels dramatically increased in the full-AD condition, thus excluding the possibility of an early diagnosis, and showing the importance of distinguishing the disease stage when designing a screening study [258]. Of note, these differences were only observed when considering the whole plasma samples, while no changes were found in free plasma and plasma-derived exosomes alone [258].

Overall, despite these promising data, more research is certainly needed before lncRNA-based AD diagnostics reach the clinic. Moreover, combinations between lncRNAs and other circulating biomarkers, as well as morphological and physical features of brain tissues, should be also considered to strengthen the results, as already suggested [293].

### 3.7. Lipids

Lipids constitute around 50% of dry brain weight and they exert key roles in basic brain functions, such as blood–brain barrier (BBB) integrity, myelination, vesicle trafficking, APP processing, and neuroinflammation [294,295]. Since alterations in these processes have been implicated in the pathophysiology of several brain disorders, including AD, a proposal has been made for their use as markers [296]. However, standard imaging methods (i.e., PET or MRI) are not suitable for lipid detection, and brain biopsies remain inapplicable, thus leaving the CSF as a potential source of biomarkers [297,298]. More recently, as changes in the circulating lipids seem to mirror the dysregulation of their profile in the brain, blood has become a viable alternative to invasive CSF sampling [299,300,301]. At the same time, the emergence of cutting-edge techniques, such as peripheral lipidomics, triple quadrupole mass spectrometry, and isobaric tagging methods, have allowed the establishment of lipid signatures potentially associated with AD, even at the prodromal and preclinical stages [302,303,304]. Lipid peroxidation, caused by oxidative stress, is one of the most-studied markers of disease, and various molecules have been proposed as potential circulating biomarkers (See Section 3.5 *Oxidative Stress*) [166,167,182,305,306,307]. Concerning fatty acids, dysregulation in their profile is linked to increased risk of dementia, with hexacosanoid acid (C26:0) being the most upregulated both in plasma and red blood cells of AD patients [303,308,309]. A correlation between the primary fatty acid amide level in plasma and Aβ pathology, hippocampal volume, and cognitive score was also reported [310]. Instead, opposite trends were observed regarding the serum content of two saturated fatty acids (palmitic and myristic acids) and three unsaturated fatty acids (oleic, linolenic, and docosahexaenoic acids), with docosahexaenoic acid being the most significantly decreased in AD compared to controls [311,312].

Phospholipids and sphingolipids have also been proposed as potential AD biomarkers [313,314]. A study conducted by Kim et al., applying mass spectrometry and ultra-performance liquid chromatography to plasma samples from 205 AD patients and 207 healthy subjects, revealed that enhanced levels of circulating ceramides (Cer16:0, Cer18:0 and Cer24:1) and phosphatidylcholines (PC36:5 and PC38:6) are associated to cognitive decline, with PC36:5 mostly correlating with the younger AD cohort [315]. Diminished concentrations of phosphatidylcholine (PC) and phosphatidylethanolamine (PE) in the serum of AD patients were also reported by other studies [316]. In particular, while decreased serum PE and enhanced lysoPE were found to predict the rate of progression from MCI to AD [314], the ratio between plasma PC aa C34:4 (a phosphatidylcholine with diacyl residue C34:4) and lysoPC a C18:2 (a lysophosphatidylcholine with acyl residue C18:2) was able to differentially diagnose MCI, AD, and healthy individuals, reaching up to 85% accuracy [317]. Interestingly, in 2014, a panel of ten peripheral-blood lipids (PCaaC36:6, PCaaC38:0, PCaaC38:6, PCaeC40:6, lysoPCaC18:2, C3, C16:1-OH, PCaaC40:2, PCaaC40:1, and PCaaC40:6) was found to predict AD or MCI onset 2–3 years in advance, with over 90% accuracy; however, these results remain to be validated by independent studies [318]. More recently, while a combination of plasma PCs (PC16:0/20:5, PC16:0/22:6, and PC18:0/22:6) has been linked to poor cognitive scores [319], a group of three serum lipid metabolites (SM (OH) C24:1, SM C24:0, and PC ae C44:3) has been demonstrated as being capable of distinguishing between early AD and MCI subjects, though no control cohort was included in the study [320].

In addition to its important role in atherosclerosis and cardiovascular disease, cholesterol has been implicated in the pathophysiology of AD and MCI [321]. For instance, a decreased plasma desmosterol/cholesterol ratio has been found to correlate with MMSE performance and the rapidity of AD progression [322]. Moreover, results from a meta-analysis comprising a total of 6127 healthy individuals and 3423 AD patients showed an inverse correlation between serum LDL, and total cholesterol levels, versus cognitive performance [323]. These data were later confirmed by further analysis [321,324]. Alternatively, among those with MCI, while similar trends were observed concerning TC, no significant differences in serum LDL were retrieved compared to healthy controls [321]. However, reproducibility issues remain, particularly concerning the criteria used for patient distribution into subgroups. For example, evidence from a cross-sectional study conducted on 1889 Chinese participants reported an inverse U-shaped association between total cholesterol levels and cognitive score only in the subgroup of patients characterized by normal levels of homocysteine [325], while Huang et al. could reach statistical significance only in late-life obese APOE-ε 4 non-carriers [326]. Furthermore, similar to the U-shaped relationship observed for total cholesterol, higher plasma HDL levels have been found in AD patients versus controls in two prospective population-based investigations [327], while very low circulating HDL content was reported to be associated with cognitive decline [328]. Given the central role of apolipoprotein E in the pathogenesis of AD [329], biomarkers based on its alteration should be investigated. In this respect, while diminished plasma ApoE was reported to predict AD development, ApoA1, ApoH, and ApoJ were found altered in MCI subjects compared to healthy controls [301,311]. Finally, serum 24-hydroxycholesterol, a marker of cholesterol metabolism in the brain [330], is decreased in AD compared to controls, with its plasma esters being lower in MCI individuals progressing towards AD than in non-progressing individuals [299,300]. However, similar findings have also been described in the context of PD, thus questioning the specificity of this biomarker [331].

Overall, these results show how promising lipid biomarkers are, especially when used in combination, but reproducibility and validation is needed before reaching any clinical application.

### 3.8. Vitamins

Vitamins are essential constituents of our diet, and are involved in many physiological and pathological mechanisms [332,333]. Hypovitaminosis is implicated in the pathogenesis of various disorders [334,335], and there is increasing evidence that vitamins also play a key role in neurodegenerative diseases, leading to hypotheses concerning their use as disease biomarkers [336,337]. Regarding AD, although preliminary data are certainly encouraging, and the role of vitamins as biomarkers for AD has been widely investigated in literature (Table 3) [188,297,338,339,340,341,342,343,344,345,346,347,348,349,350,351], some limitations in terms of consistency, reproducibility, and specificity remain to be solved, and further research is needed before hypothesizing a clinical application.

#### 3.8.1. Water-Soluble Vitamins: Vitamins B and C

Several studies have shown the importance of B vitamins for proper physiological and neurological functioning [334]. Concerning AD, some associations were found between low plasma levels of vitamin B_1_ (thiamine) and AD development [352], with high levels of its active form (thiamine diphosphate) being reported to be a protective factor against neurodegeneration [340]. Similarly, insufficient dietary intake of vitamin B_2_ (riboflavin) was associated with an augmented incidence rate of AD in patients not presenting the APOE-ε 4 genotype [341]. Interestingly, more significant changes in both vitamin B_1_ and B_2_ levels have been described in AD women compared to men, thus showing the importance of considering gender differences when designing a large-cohort study [340,341].

High concentrations of homocysteine in the blood (hyperhomocysteinemia) have been linked to cardiovascular and neurological disorders [353]. In this respect, vitamin B_6_ supplementation was observed to lower homocysteine levels in Aβ_1–42_-treated PC12 cells and in the brains of APP/PS1 mice, thus exerting a positive impact on an established biomarker for AD [164]. However, while a study involving 2533 participants showed a link between reduced cognitive decline and appropriate intake of vitamins B_9_ (folate), B_6_, and B_12_ [342], no significant differences were reported in another study comprising 202 AD patients when accounting for age, gender, education, and other covariates [354]. Nonetheless, a small study conducted on 48 patients followed up after 7–9 years showed a diminished amount of folate in the blood of MCI patients converting to AD [297]. When using machine learning approaches, the concomitant findings of reduced blood levels of folic acid and vitamin D were confirmed to be predictive of worse MMSE scores (that is, a more pronounced cognitive impairment), thus suggesting a possible link with disease severity [343]. Moreover, diminished levels of circulating vitamin B_12_ in patients with AD were reported in another recent case-control study [344]. Interestingly, vitamin B_12_ and total tau plasma levels were inversely related in a longitudinal study, thus confirming the correlation between vitamins and AD pathogenesis [345]. Again, this association was stronger for women, who might have a higher probability than males to convert to AD [355,356]. Despite these promising data, however, the role of vitamin B_12_ as a possible biomarker is far from being established. A recent study analyzing data from the ADNI cohort and the Australian Imaging, Biomarker & Lifestyle Flagship Study of Ageing (AIBL) has shown contradictory results regarding the impact of vitamin B_12_ on neurological functions, probably depending on the clinical condition [357].

Similar to B vitamins, some evidence suggests that blood concentrations of vitamin C are decreased in AD patients compared to controls, thus representing another possible disease biomarker [351].

#### 3.8.2. Liposoluble Vitamins: Vitamins D, A, and E

Over time, low concentrations of vitamin D were observed to be linked to neurodegeneration, and to a greater likelihood of cognitive impairment and dementia [346,358,359]. In particular, low 25(OH)D_3_, unlike 1,25(OH)_2_D_3_, was demonstrated to be remarkably associated with MCI and AD [188,347]. Similarly, it has been observed that 25(OH)D_3_ in the plasma might correlate with Aβ_1–42_ in CSF and cognitive status, thus linking blood and CSF biomarkers [348,360]. Furthermore, an augmented plasma concentration of 25(OH)D_3_ was linked to more pronounced cerebral volumes, especially white matter and medial temporal lobe structures, while decreased 25(OH)D_3_ in serum was associated with deficits in brain connectivity and smaller hippocampal sizes in MCI subjects [348,349,361]. Interestingly, if both genotype and vitamin D levels were considered in the same study, it has been found that, among late-onset AD patients, 25(OH)D_3_ insufficiency was observed only in ApoE-ɛ 4 non-carriers, thus suggesting the use of vitamin D as a possible disease biomarker only in this category of patients [346,362]. Nonetheless, serum levels of vitamin D remain a controversial candidate as an AD biomarker; indeed, unstandardized data and inconsistency among analytical methods still present an issue, and further evidence is needed to clarify the role of 25(OH)D_3_ serum levels in MCI and AD before considering its clinical application [363].

Regarding vitamins A and E, while data from a cross-sectional study reported decreased blood concentrations of vitamin A in AD participants [351], in another study, serum deficiency of both vitamin A and E was correlated with cognitive impairment in patients with dementia other than Alzheimer’s [350], thus emphasizing that additional studies are required to better assess both the specificity and consistency of these potential biomarkers.

### 3.9. Gut Microbiota

Gut microbiota alterations have been reported in several different conditions, including neurodegeneration and AD [364,365,366,367,368,369,370,371,372,373,374]. Overall, AD patients seem to be characterized by dysbiosis, a condition of bacterial imbalance with a predominance of pro-inflammatory taxa and a decrease in beneficial anti-inflammatory species [375,376,377,378]. Dysbiosis is normally accompanied by an altered gut immune response, favoring epithelial cell leakage, increased bacterial translocation, and enhanced systemic inflammation [379]. Though data are still inconclusive, gut bacteria family and genus shifts might represent promising tools as novel biomarkers for the diagnosis and progression of AD, and the restoration of gut microbiota balance a potential therapeutic approach [380]. Several bacterial metabolites are already used as fecal biomarkers to characterize dysbiosis in AD patients; for instance, higher levels of trimethylamine N-oxide (a microbial metabolite implicated in immune response activation), enhanced oxidative stress, and intestinal barrier dysfunction have been identified in MCI and AD patients compared to cognitively unimpaired individuals [381,382].

Some microbiota-derived molecules present in the systemic circulation have also been considered as potential blood biomarkers for AD. For example, increased levels of circulating LPS, the main component of the outer membrane in Gram-negative bacteria, have been identified in MCI and AD patients [383]. Indeed, LPS can induce the activation of pro-inflammatory pathways, promoting BBB disruption and neuroinflammation [384,385]. Furthermore, short-chain fatty acids (SCFAs), usually beneficial for their anti-inflammatory and antioxidant properties in the intestinal lumen, can be released in the blood upon dysbiosis, reach the cerebral circulation, and cause potentially harmful effects on brain function [383,386,387]. Moreover, given that gut dysbiosis may induce neuroinflammation, other inflammatory factors, such as IL-6 and INF-γ, have also been considered as potential AD biomarkers [386].

Although blood biomarkers are non-invasive and rapid diagnostic tools, and the preliminary results appear promising, several limitations still exist, making microbiota-derived blood biomarkers inconclusive, and not yet effective tools for AD diagnosis. Indeed, large-cohort studies are needed, ideally considering a combination of several biomarkers, which include bacterial composition and microbiota-derived metabolites. Additionally, since gut microbiome composition and function are affected by lifestyle, age, gender, dietary intake, and geography, it is important to at least limit all the possible confounding factors that can influence study results [388,389]. Overall, the increasing research on the role of gut microbiota in disease pathogenesis and progression could also prove useful in the discovery of novel minimally invasive biomarkers, although more studies are still required to better address this point.

## 4. Discussion

As confirmed by numerous scientific evidence, the neuropathology associated with AD is already traceable many years before clinical onset. For this reason, the study of the preclinical phases, that is, of cognitively healthy subjects at risk of developing dementia due to the presence of the neuropathological signs of AD, is of particular importance. In 2018, the National Institute on Aging (NIA) and the Alzheimer’s Association (AA) proposed a new clinically unbiased classification system of the disease based on the presence (or absence) of three processes: amyloidosis, tauopathy, and neurodegeneration (ATN classification); detectable by examination of the CSF, and via PET and MRI [390,391]. The ATN classification thus identifies eight possible risk profiles for AD, from completely negative A − T − N − to completely positive A + T + N +. At present, however, it is not known which of these profiles is associated with an increased risk of AD or cognitive decline. A first possible answer to this question comes from a recent study that combined the data of four cohorts, for a total of 814 participants, followed for an average follow-up period of 7 years, and classified according to the ATN scheme [392,393]. The results revealed that only subjects classified as A + T + N + show marked cognitive impairment compared to subjects classified as A − T − N −. The same data emerged using a previous classification of the NIA–AA group, based on the presence (or absence) of amyloidosis and tau, leading to the conclusion that the concomitant presence of amyloidosis and tau pathology is required to increase the risk of developing cognitive impairment in the future [394]. However, the high invasiveness and the elevated costs of CSF sampling, as well as the imaging methods, have recently led scientists to search for new minimally invasive and cost-effective blood-based biomarkers to be used in broad population screenings [11]. In this respect, the intrinsic multifactorial etiology of AD offers the possibility to search for a large number of biomarkers belonging to different categories. The application of these biomarkers in AD diagnosis and prognosis ranges from common bench tests to molecular biology; therefore, their affordability depends on the goals the biomedical expert aims to reach. Proteomics [395] and transcriptomics [396] are to be considered fundamental in discovering and understanding the complex correlations of the many active biomarkers with brain pathology; for example, the mitochondrial signature of AD [397]. The most promising research concerns new possible molecular biology techniques with which early diagnosis can be made. AD is a disease that has a very slow development process: the obvious dementia symptoms are the tip of the iceberg of brain changes, while the “invisible” biological correlates in the AD subject start up to 20 years earlier. It is evident that, without a correct diagnosis and without knowing why these changes occur (why do some proteins, such as beta-amyloid and tau, accumulate? And are these the cause of the disease, or a consequence of it?), the pharmacological approach may be ineffective and imprecise, relying on a symptomatic approach. Therefore, the future of research is focusing on techniques that allow abnormalities to be identified before they are irreversible.

The affordability, feasibility, and cost-effectiveness of many molecular biology kits and assays, which should enable physicians to diagnose AD at the earliest stage, have to be compared with the huge costs of caring for the AD patient. From worldwide estimates, ADI (Alzheimer’s Disease International) reported over 9.9 million new cases of AD-caused dementia per year in 2015, that is, a new case every 3.2 s.

In this narrative review, we summarized the main findings regarding dysregulations in lipids, metabolites, oxidative stress, inflammation, gut microbiota, vitamins, and non-coding RNAs in AD patients compared to controls. The huge amount of data and evidence reported in this paper, however, may lack sufficient elaboration to allow the reader to grasp the overriding value of the enormous amount of data reported in the results. This is not only a limitation of our extensive review, which should gather as many novelties in the field as possible, but it represents a weakness of the AD research worldwide, overinflated with the enormous crowding of biomolecular data, yet showing scant ability in using this data as an orchestrated methodology to narrow the time between earliest symptoms or signs and diagnosis. A recent systematic review by van der Schaar et al. proposed that a starting point for clinicians is to deepen the discussion about biomarkers, more than personal views or thoughts from societal contexts, particularly to diagnose AD before dementia [398]. This should make this review particularly important to accurately know what is currently discussed in the neurobiology of AD diagnosis.

However, several limitations still exist and need to be addressed before clinical application. First, as broadly discussed in the text, specificity remains a concern. Hypovitaminosis, oxidative stress, ncRNAs fluctuations, high levels of pro-inflammatory cytokines and systemic inflammation, alterations in metabolic and lipidomic profiles, and dysbiosis are common to many different conditions [399,400,401,402,403,404,405]. Second, studies including age- and gender-matched cohorts should be preferred, as physiological alterations in fluid biomarkers have been reported during aging and between males and females [406]. Of note, more advanced biomarkers with the potential for clinical application do not seem exempt from age and sex impact, as demonstrated by recent investigations from the APMI and the INSIGHT-preAD study [345].

Interpersonal changes due to comorbidities, genetic background, and lifestyle should also be accounted for, and, in this respect, studies with very large numbers of participants are encouraged [407,408,409]. Moreover, the use of standardized tests, shared inclusion criteria, and consistent statistical analysis are of major importance to ensure reproducibility, as often independent studies are not able to replicate previous data, thus limiting clinical advancement [410,411].

Furthermore, the recent introduction of machine learning (ML) for the diagnosis of AD and the prediction of MCI, represents an advancement in the availability of tools able to reach high performance in AD diagnosis [412,413]. In this respect, ML can support the diagnostic investigation of MCI progression from the metabolic signature pattern [414].

Yet, some particularly advanced and cutting-edge techniques, such as peripheral lipidomics, triple quadrupole mass spectrometry, and isobaric tagging methods, are particularly burdensome for clinical routine analysis, and here were described for completeness, whereas others are very rarely applied [302,303,304].

Lastly, as several authors mainly focus on a single molecule, it would be interesting to investigate whether a combination of multiple biomarkers from different categories could strengthen early diagnostic accuracy, potentially offering the opportunity to establish distinct panels of biomarkers for distinct stages of AD onset and progression.

Overall, although promising data have been recently reported, more research is required to ensure the specificity, sensitivity, cost-effectiveness, and reproducibility of blood-based AD biomarkers, with the ultimate goal of helping diagnosis and improving therapy.

## 5. Conclusions

Despite the vast amount of data on novel AD biomarkers, medicine is still getting to grips with the full extent of bioanalytical and imaging tools to diagnose AD at its earliest stage, and to differentiate AD from other cognitive impairments and neurodegenerative conditions. This report attempts to provide a thorough review of the many different kinds of biomarkers for AD that are being studied, and that shed light on the fundamental and diverse pathological mechanisms of AD. These insights from scientific bench studies can hopefully be translated into clinical diagnostics and medical therapeutics for this devastating disease.

## Figures and Tables

**Table 1:** Oxidative-stress-related blood AD biomarkers.

Ref	Study Cohort and Design	Plasma/Serum	Measurement Methods	Results	Cohort of Variation	Biomarker/s Proposed
Han et al., 2021 [164]	Aβ_1–42_-treated PC12 cells, brain and hippocampus of APP/PS1 mouse, and the serum of AD patients	Serum	Probe 1 *, ELISA assay or LC–MS	↑ Hcy ↓ Cys and GSH	AD vs. HC	Hcy, Cys, and GSH changes in the serum
Evlice et al., 2017 [165]	30 AD (15 females and 15 males) and 10 HC (7 males and 3 females)	Serum	Activity and quantitative G6PD kit	↑ serum G6PD	AD vs. HC	Serum G6PD levels
Peña-Bautista et al., 2021 [166]	12 preclinical AD and 31 HC	Plasma	Chromatography and mass spectrometry	↓ lipid peroxidation -15-F_2t_-IsoP correlates with p-tau -15-F_2t_-IsoP correlates with t-tau	AD vs. HC (non-significant)	Plasma isoprostanoids (combination of 10 biomarkers)
Zengi et al., 2012 [168]	21 AD (10 men and 11 women) and 20 HC (11 men and 9 women)	Serum	PON1 activity absorbance assay	↓ serum PON1	AD vs. HC	Serum PON1 activity
López et al., 2013 [176]	36 AD, 18 MCI, and 33 aged HC	Blood		↑ Copper and MDA	AD and MCI vs. HC	Blood copper, MDA, and SOD
Pradhan et al., 2022 [175]	47 AD, 43 MCI, and 48 HC	Serum	SPR and Western blot	↓ SIRT1, SIRT3, and SIRT6	AD vs. MCI and HC	Serum SIRT1, SIRT3, and SIRT6 concentration
Cardoso et al., 2014 [170]	27 AD, 17 MCI, and 28 HC	Plasma	Hydride generation atomic absorption spectroscopy	↓ plasma Se ↓ erythrocyte Se	-AD vs. MCI and HC -AD and MCI vs. HC	Plasma Se levels
García et al., 2021 [10]	20 MCI (13 males and 7 females), 20 AD (11 males and 9 females), and 15 PD (12 males and 3 females) and HC (age and sex matched).	Plasma	Electrochemical immunosensor	↑ Unfolded p53 ↑ Unfolded p53	-MCI, AD, and PD vs. HC -AD vs. MCI and PD	Plasma unfolded p53
Peña-Bautista et al., 2021 [167]	6 AD and 13 MCI	Plasma	LC–MS	↑ dihomo-isoprostanes (17-epi-17-F2t-dihomo-IsoP, 17-F2t-dihomo-IsoP, Ent-7(RS)-7-F2t-dihomo-IsoP) and neuroprostanes (10-epi-10-F4t-NeuroP)	AD vs. MCI	Plasma isoprostanoids levels
Picco et al., 2014 [169]	23 SCI, 28 MCI, and 34 mild AD	Plasma	Spectrophotometric analysis	↓ eSOD activity ↓ CAT activity = GPx activity	-AD vs. SCI -AD vs. MCI and SCI	Plasma eSOD, CAT, and GPx activity combined with functional neuroimaging
Lin et al., 2021 [163]	49 MCI and 16 HC	Plasma	Commercially available assay kit	↓ plasma GSH	MCI vs. HC	Plasma GSH levels
Li et al., 2021 [173]	839 HC	Serum		↑ Serum uric acid = Serum uric acid in healthy individuals with or without tau pathology	Preclinical AD vs. HC	Serum uric acid
Du et al., 2019 [172]	113 aMCI and 832 HC	Serum	Commercial ELISA kit	↑ Serum IMA and IMA/albumin	aMCI vs. HC	Serum IMA
Wu et al., 2021 [171]	88 HC, 201 with cognitive impairment and no dementia (CIND) and 207 with dementia (160 AD and 47 vascular dementia)	Plasma	LC–MS/MS	↓ plasma	Dementia vs. CIND and HC	Plasma ergothioneine levels

Abbreviations: AD, Alzheimer’s disease; aMCI, amnestic mild cognitive impairment; APP/PS1, double transgenic mouse model of AD; CAT, catalase; ELISA, enzyme-linked immunosorbent assay; eSOD, extracellular superoxide dismutase; G6PD, glucose-6-phosphate dehydrogenase; GPx, glutathione peroxidase; GSH, glutathione; HC, healthy controls; Hcy, homocysteine; IMA, ischemia-modified albumin; LC–MS, liquid chromatography–mass spectrometry; MCI, mild cognitive impairment; MDA, malondialdehyde; PD, Parkinson’s disease; PON1, paraoxonase 1; SCI, subjective cognitive impairment; SEC–ICP–MS, size exclusion chromatography–inductively coupled plasma–mass spectrometry; SPR, surface plasmon resonance; * Probe 1, ethyl (E)-3-(9-chloro-11-oxo-2,3,6,7-tetrahydro-1H,5H,11H-pyrano [2,3-f] pyrido [3,2,1-ij] quinolin-10-yl)-2-cyanoacrylate; ↓, decrease; ↑, increase.

**Table 2:** Circulating ncRNAs as AD biomarkers.

Ref	Study Cohort	Plasma/ Serum/Blood	Upregulated	Downregulated	Cohort of ncRNA Variation	Method
Dakterzada et al., 2021 [252]	Discovery cohort (*n* = 19, mild AD) and validation cohort (*n* = 53, mild AD)	Plasma		miR-342-5p	Severe AD	RT–PCR
Poursaei et al., 2022 [213]	50 AD and 50 HC	Plasma	hsa-let7d-5p hsa-let7g-5p		AD	RT–PCR
Galimberti et al., 2014 [232]	22 AD, 18 NINDCs, 8 NIDCs, and 10 FTD	Serum		miR-125b miR-23a miR-26b	AD	RT–PCR
Kumar et al., 2017 [235]	Discovery cohort (10 AD, 6 MCI, and 14 HC) and validation cohort (11 AD, 20 MCI, and 18 HC)	Serum	miR-455-3p miR-4668-5p		AD	RT–PCR
Yilmaz et al., 2016 [223]	172 AD and 109 HC	Whole blood		hsa-miR-9-5p hsa-miR-106a-5p hsa-miR-106b-5p hsa-miR-107	AD	RT–PCR
Wang et al., 2020 [244]	120 AD, 120 PD, and 120 HC	Plasma	miR-107	miR-103	AD	RT–PCR
Barbagallo et al., 2020 [259]	30 AD, 30 PD, 24 VD, 25 VP, and 30 HC	Serum	miR-22 miR-23a miR-29a miR-125b		AD	RT–PCR
Fotuhi et al., 2019 [258]	45 AD and 36 HC	Whole plasma	lncRNA BACE1-AS		AD	RT–PCR
Feng et al., 2018 [257]	88 AD and 72 HC	Plasma	lncRNA BACE1		AD	RT–PCR
Yang et al., 2015 [222]	30 AD and 30 HC	Blood		miR-29c	AD	RT–PCR
Bhatnagar et al., 2014 [233]	110 AD and 123 HC	Plasma	miR-34c		AD	RT–PCR
Leidinger et al., 2013 [17]	106 AD, 18 MCI, 16 CIS, 9 PD, 15 DEP, 15 BD, 14 SCHIZ, and 22 HC	Blood	hsa-miR-30d-5p	hsa-miR-144-5p	AD	NGS and RT–PCR
Zhu et al., 2015 [256]	26 AD, 30 MCI, and 42 HC	Serum		miRNA-210	AD	RT–PCR
Kiko et al., 2014 [211]		Plasma		miR-34a miR-146a	AD	RT–PCR
Xing et al., 2016 [226]	30 AD and 30 HC	Blood	miR-206		AD	RT–PCR
Wu et al., 2020 [227]	40 AD (amyloid positive) and 31 controls (amyloid negative)	Blood		miR-146b-5p miR-15b-5p	AD	Small RNA sequencing
Kumar et al., 2013 [210]	11 AD, 9 MCI, and 20 HC	Plasma		hsa-miR-191-5p hsa-miR-15b-5p hsa-let-7d-5p hsa-let-7g-5p hsa-miR-142-3p	AD	nCounter miRNA expression assay v1 and RT–PCR
Geekiyanage et al., 2012 [220]	7 AD and 7 HC	Serum		miR-137 miR-181c miR-9 miR-29a/b	AD	RT–PCR
Tan et al., 2014 [72]	105 AD and 150 HC	Serum	miR-9	miR-125b miR-181c	AD	RT–PCR
Sørensen et al., 2016 [212]	10 AD and 10 VD/FTD or LBD	Plasma	miR-590-5p miR-142-5p	miR-194-5p	AD	RT–PCR
Ludwig et al., 2019 [225]	AD, MCI, HC, and ODN (total subjects 465)	Blood		miR-532-5p	AD	RT–PCR
Liu et al., 2014 [247]	32 MCI, 45 AD, and 50 HC	Serum		miR-384	AD	RT–PCR
Wang et al., 2019 [218]	7 AD and 5 HC	Plasma		miR-200a-3p	AD	Microarray miRNA profile
Liu et al., 2020 [224]	50 AD, 20 VD, and 50 HC	Blood	miR-574-5p	hsa-circ-0003391	AD	Microarray analysis
Hara et al., 2017 [234]	27 AD and 18 HC	Serum		hsa-miR-501-3p hsa-let-7f-5p hsa-miR-26b-5p	AD	RT–PCR
Jia et al., 2016 [231]	84 AD and 62 HC	Serum	miR-519	miR-29, miR-125b, miR-223	AD	RT–PCR
Cosín-Tomás et al., 2017 [249]	HC, AD, PAD (*n* = 35 per group), and PD (*n* = 20)	Plasma		miR-34a-5p miR-545-3p	AD	RT–PCR
Nagaraj et al., 2017 [248]	15 MCI, 20 AD, and 15 HC	Plasma	miR-483-5p miR-486-5p miR-200a-3p miR-142-3P	miR-30b-5p	AD and MCI	RT–PCR
Dong et al., 2015 [242]	127 AD, 30 MCI, and 30 VD	Serum	miR-93 miR-146a	miR-31 miR-93 miR-143 miR-146a	AD and MCI	Solexa sequencing and RT–PCR
Siedlecki-Wullich et al., 2019 [251]	56 AD, 26 MCI, 38 HC, and 27 FTD	Plasma	miR-92a-3p miR-181c-5p miR-210-3p		AD and MCI	RT–PCR
Sabry et al., 2020 [246]	40 MCI and AD, and 20 HC	Plasma	miRNA-483-5p		AD and MCI	RT–PCR
Zhang et al., 2021 [254]	75 MCI and 52 HC	Serum	hsa-let-7g-5p hsa-miR-107 hsa-miR-186-3p		MCI	RT–PCR
Shi et al., 2020 [253]	71 aMCI and 69 HC	Serum	miR-34c		aMCI	RT–PCR
He et al., 2021 [250]	Discovery cohort (*n* = 10), analysis cohort (*n* = 30), and validation cohort (*n* = 80)	Plasma		miR-1185-2-3p miR-1909-3p miR-22-5p miR-134-3p	aMCI	Microarray sequencing
Wang et al., 2015 [255]	97 AD, 116 aMCI, and 81 HC	Plasma		miR-107	aMCI	RT–PCR

Abbreviations: AD, Alzheimer’s disease; aMCI, amnestic mild cognitive impairment; BD, bipolar disorder; CIS, clinically isolated syndrome; DEP, major depression; FTD, frontotemporal dementia; HC, healthy controls; INDCs, inflammatory neurological controls; LBD, Lewy body dementia; MoCA, Montreal Cognitive Assessment; NINDCs, non-inflammatory neurological controls; PAD, preclinical AD; PD, Parkinson’s disease; SCHIZ, schizophrenia; VD, vascular dementia; VP, vascular parkinsonism.

**Table 3:** Vitamin-based biomarkers for AD.

Ref	Study Cohort and Design	Analysis Performed	Results	Cohort of Variation	Biomarker/s Proposed
Glasø et al., 2004 [338]	AD (*n* = 20), HC (*n* = 18)	Analysis on serum and blood	↓ Blood thiamine ↓ Blood TDP	AD	Vit B_1_
dos Santos et al., 2020 [344]	AD (*n* = 60), HC (*n* = 60)	Complete blood count and Vit B_12_ levels assessment	↓ Vit B_12_	AD	Vit B_12_
Lanyau-Domínguez et al., 2020 [351]	AD (*n* = 43), MCI (*n* = 131), HC (*n* = 250)	Spectrophotometry and high-resolution liquid chromatography on plasma	↓ Vit A and vit C	AD	Combination of vit A and vit C
Gold et al., 1995 [339]	AD (*n* = 17), n-AD (*n* = 17)	Microbiologic assay on plasma and RBC	↓ Plasma thiamine -No correlation between RBC thiamine and AD	AD	Vit B_1_
Wang et al., 2018 [340]	AD (*n* = 90), HC (*n* = 90)	HPLC on whole blood samples	↓ TDP	Female AD vs. male AD	TDP as protective factor for AD
D’Cunha et al., 2019 [341]	AD (*n* = 63), HC (*n* = 63)	ELISA kit to determine APOE4 on serum	↓ Vit B_2_ dietary intake	AD without APOE4 genotype	Vit B_2_ and folate
Dursun et al., 2016 [346]	EOAD (*n* = 22), LOAD (*n* = 72), MCI (*n* = 32), HC (*n* = 70)	Chemiluminescent immunoassay on serum	↓ 25(OH)D	LOAD ApoEε4 non-carriers	Vit D (in ApoEε4 allele non-carriers)
Ouma et al., 2018 [347]	AD (mild: *n* = 41, moderate: *n* = 35, severe: *n* = 32), MCI (*n* = 61), HC (*n* = 61)	Competitive radioimmunoassay on serum	↓ 25(OH)D_3_	MCI and AD	25(OH)D_3_
Blasko et al., 2021 [297]	Non-converting HC (*n* = 13), HC converting to MCI (*n* = 6), HC converting to AD (*n* = 6), MCI converting to AD (*n* = 8), MCI converting to HC (*n* = 8) and stable MCI (*n* = 7)	Competitive immunoassay on serum	↓ Folate	MCI–AD converting pt	Folate
An et al., 2019 [342]	2533 participants followed for an average of 2.3 y	Immunoassay on serum	↑ Folate, vit B_6_, and vit B_12_ intake	Pt with better cognitive reserve	B vitamins and folate
Murdaca et al., 2021 [343]	AD (*n* = 108)	Machine learning approach to correlate blood vitamin levels with MMSE score	↓ Vit D and folic acid	Pt with lower MMSE score	Combination of vit D and folic acid
Baldacci et al., 2020 [345]	SMC (*n* = 316)	Aβ-PET (*n* = 316, at baseline and 2 y follow-up). Lumbar puncture (*n* = 40 at baseline). Immunoassay on plasma (*n* = 79, at baseline, 1 y and 3 y follow-up)	↓ Vit B_12_	Pt with higher plasma total Tau levels	Vit B_12_
de Leeuw et al., 2020 [188]	SCD (*n* = 149), MCI (*n* = 150).	Analysis on serum and plasma	↑ 1,25(OH)_2_D_3_	SCD	1,25(OH)_2_D_3_
Hooshmand et al., 2014 [348]	AD (*n* = 18), MCI (*n* = 28), SCI (*n* = 29)	Immunoassay on plasma, ELISA on CSF, MRI scans	↑ 25(OH)D_3_ ↑ 25(OH)D_3_	-Pt with higher CSF Aβ_1–42_ levels -Pt with greater brain volumes	Vit D
Al-Amin et al., 2019 [349]	MCI (*n* = 54)	Analysis on serum MRtrix and NBS on MRI scans	↓ 25(OH)D_3_	Pt with reduction in total hippocampal volume and connection deficit	Vit D
Raszewski et al., 2015 [350]	AD (*n* = 33), n-AD (*n* = 31)	HPLC on serum	↓ Vit A and vit E	n-AD	Combination of vit A and vit E

Abbreviations: AD, Alzheimer’s disease; CSF, cerebrospinal fluid; ELISA, enzyme-linked immunosorbent assay; EOAD, early-onset AD; HC, healthy controls; HPLC, high-performance liquid chromatography; LOAD, late-onset AD; MCI, mild cognitive impairment; n-AD, non-Alzheimer’s dementia; NBS, network-based statistic; pt, patients; RBC, red blood cells; SCD, subjective cognitive decline; SCI, subjective cognitive impairment; SMC, subjective memory complaints; TDP, thiamine diphosphate; y, years; ↓, decrease; ↑, increase.

## Data Availability

Not applicable.
